# Cancer-associated fibroblasts—heroes or villains?

**DOI:** 10.1038/s41416-019-0509-3

**Published:** 2019-07-10

**Authors:** Krystyna A. Gieniec, Lisa M. Butler, Daniel L. Worthley, Susan L. Woods

**Affiliations:** 1 School of Medicine, University of Adelaide, Adelaide, SA Australia; 2 Precision Medicine, South Australian Health and Medical Research Institute, Adelaide, SA Australia

**Keywords:** Cancer microenvironment, Cancer microenvironment, Cancer microenvironment

## Abstract

Cancer-associated fibroblasts (CAFs) were originally presumed to represent a homogeneous population uniformly driving tumorigenesis, united by their morphology and peritumoural location. Our understanding of CAFs has since been shaped by sophisticated in vitro and in vivo experiments, pathological association and, more recently, ablation, and it is now widely appreciated that CAFs form a group of highly heterogeneous cells with no single overarching marker. Studies have demonstrated that the CAF population contains different subtypes based on the expression of marker proteins with the capacity to promote or inhibit cancer, with their biological role as accomplices or adversaries dependent on many factors, including the cancer stage. So, while CAFs have been endlessly shown to promote the growth, survival and spread of tumours via improvements in functionality and an altered secretome, they are also capable of retarding tumorigenesis via largely unknown mechanisms. It is important to reconcile these disparate results so that the functions of, or factors produced by, tumour-promoting subtypes can be specifically targeted to improve cancer patient outcomes. This review will dissect out CAF complexity and CAF-directed cancer treatment strategies in order to provide a case for future, rational therapies.

## Background

Fibroblasts are non-specialised cells of the connective tissue stroma that support tissue function and homoeostasis by regulating the extracellular matrix (ECM), inflammation and epithelial cell proliferation and differentiation (reviewed in ref. ^1^) They are predominantly quiescent, only transiently acquiring activity during periods of tissue remodelling and repair. Activated fibroblasts will then either apoptose or revert back to a dormant state once their function becomes redundant; otherwise, pathological conditions such as tissue fibrosis and chronic inflammation can arise.^2^ This chronic tissue repair response has been implicated in the progression to cancer,^3^ and tumours can therefore be considered as ‘wounds that do not heal’.^4^ Distinct, activated fibroblasts that surround tumours are termed cancer-associated fibroblasts (CAFs) and they, along with other elements of the tumour microenvironment, can indulge in reciprocal dialogues with the neoplastic epithelial cells to maximise tumour fitness.

The role of CAFs in cancer has risen to prominence, with genes positively associated with colorectal cancer (CRC) recurrence and poor prognosis predominantly found to be upregulated within the CAF population, not in tumour cells.^5,6^ Furthermore, when CRC was classified into four distinct consensus molecular subtypes (CMSs), it was revealed that CMS4, with over-representation of the stromal signature, was associated with more aggressive tumour stages and worse overall survival than other subtypes.^7^ CAFs have subsequently been implicated in different stages of cancer development, from primary growth to secondary colonisation at metastatic sites, in different cancer types. However, a number of contradictory studies have proposed an anti-tumour role for CAFs, as ablation of CAFs expressing specific markers, such as α-smooth muscle actin (α-SMA), or inhibition of signalling pathways proposed to be important in CAF-mediated tumorigenesis (such as hedgehog, Hh) actually resulted in more aggressive pancreatic ductal adenocarcinoma (PDAC).^8,9^ While studies involving tumour-suppressive CAF populations are in the minority, they clearly demonstrate the need to more fully understand the role of discrete and possibly opposing CAF subpopulations in order to effectively target tumours.

The identification of markers that define CAF subsets is in its infancy, and this has hindered the study of specific CAFs. Two commonly used molecular CAF markers are α-SMA and fibroblast activation protein (FAP), but these markers do not perfectly demarcate precisely the same population of cells.^10^ Many other markers for CAFs have been identified, such as vimentin, fibroblast-specific protein-1 (FSP1), and platelet-derived growth factor receptor-β (PDGFRβ),^11^ which are discussed below. It has become apparent that CAFs form a large heterogeneous population of cells expressing a wide array of molecular markers, which are not necessarily unique to fibroblasts. It might be that particular CAF subsets have a tendency for tumour promotion or suppression, perhaps determined by tumour type and location, but the biological programmes influencing these phenotypes currently remain unknown. In order to successfully use CAFs as a form of cancer treatment, CAF complexity must be resolved such that specific pro-tumorigenic subtypes can be inhibited or converted back into a quiescent state.

This review will provide an overview of our current knowledge of the pro- and anti-tumour roles of CAFs in various tumour entities and the implications for these findings on cancer treatment.

## The tumour-promoting CAF phenotype

The primary role of activated fibroblasts is to remodel and regenerate tissues, which they do under normal circumstances in a highly regulated manner. However, this function can be hijacked and enhanced in cancer, generating misregulated, tumour-promoting CAFs. As will be discussed below, CAFs can indirectly support tumorigenesis by encouraging an oxygen-rich, immunosuppressive and pro-inflammatory microenvironment. In addition, CAFs are capable of directly supporting tumour growth, invasion and metastasis.

### CAFs encourage the development of a tumour-promoting stroma

Although CAFs are the predominant cell type in the tumour stroma, many other stromal cells contribute to carcinogenesis. These include pro-inflammatory, immunosuppressive and endothelial cells, which can be recruited or influenced by CAFs via a wide array of CAF-derived factors.

Multiple studies have emphasised the tumour-promoting role of inflammatory cells, from their production of many pro-tumour factors to their mutagenic involvement in tumour evolution.^12^ CAFs can adopt a pro-inflammatory gene signature at an early stage in cancer development, induced by tumour-derived interleukin (IL)-1β and, as such, themselves mediate this tumour-enhancing inflammation.^13^ CAFs can additionally be involved in tumour immune evasion, inducing an immune-suppressive microenvironment to facilitate tumour escape from anti-tumour immune surveillance. When FAP^+^ stroma was specifically ablated using diphtheria toxin in genetically modified Lewis lung cancer (LCC) mice, cytotoxic T-cell activity was restored and rapid tumour necrosis resulted.^14^ Combining this ablation with the chemotherapeutic agent, doxorubicin, resulted in a reduced infiltration of immune-suppressor cells such as T-regulatory (Treg) cells and further activated the anti-tumour immune response.^15^ CAFs can directly deactivate the immune system, with some CAF subsets shown to express the negative co-regulatory immune signals, programmed death-ligand (PD-L)1 and PD-L2 or secrete immune-suppressive factors, such as prostaglandin E2 (PGE2), to directly reduce the activation of T and natural killer cells.^2,16^ The CAF-rich peritumoural stroma can also physically prevent the immune system from effectively interacting with the tumour. This phenomenon of immune exclusion from tumours has been linked to the lack of patient response to PD-L1 blockade in many cancers, including metastatic urothelial cancer, and it is associated with a signature of transforming growth factor-β (TGF-β) signalling in fibroblasts,^17^ which is known to activate fibroblasts and induce the dense stromal reaction. When mouse models of immune-excluded breast or metastatic CRC were co-treated with TGF-β and PD-L1 inhibitors, the expression of matrix-remodelling factors in CAFs was reduced and T cells were then able to penetrate both primary and metastatic tumour masses to cause regression.^17,18^ However, the consequences of attempts to manipulate the immune system for cancer treatment are not always predictable due to the complex interplay between different cell types in the tumour microenvironment, including fibroblasts. For example, use of a colony-stimulating factor 1 receptor (CSF1R) inhibitor for targeting pro-tumour, tumour-associated macrophages in mouse models of LLC and melanoma resulted in the enhanced recruitment of polymorphonuclear myeloid-derived suppressor cells (PMN-MDSC). The block in CSF1 signalling caused CAFs to secrete the PMN-MDSC chemokine, C–X–C Motif Chemokine Ligand 1 (Cxc11); CAFs were therefore able to neutralise the anti-tumour effect of the CSF1R inhibitor. Tumours were reduced when tumour-bearing mice were treated with both the CSF1R inhibitor and a C–X–C Motif Chemokine Receptor 2 (CXCR2) antagonist (to prevent PMN-MDSC migration), and tumours were completely blocked when a PD-1 antibody was added to this combination.^19^

CAFs can respond to hypoxia by upregulating hypoxia-induced angiogenesis regulator (HIAR), which in turn increases CAF motility and secretion of vascular endothelial growth factor A (VEGFA).^20^ This induces angiogenesis to improve tumour oxygenation, nutrient flow and waste removal, and the resultant decline in hypoxia stimulates further CAF activation.^21^ CAFs can also be influenced to stimulate a vessel-rich environment by tumour cells. As an example, hepatocellular carcinoma (HCC)-derived exosomal miRNA-21 activates hepatic stellate cells into CAFs by blocking PTEN, causing the CAFs to upregulate angiogenic cytokines including VEGF and increasing vessel density in HCC patients.^22^

### CAFs improve tumour energy synthesis

There are many ways in which CAFs can increase the net energy production of tumours, fuelling growth. The ‘reverse Warburg effect’ was recently termed^23^ to define the phenomenon whereby CAFs undergo autophagy and mitophagy, resulting in aerobic glycolysis and the release of energy-rich metabolites that are then absorbed by cancer cells to enter the tricarboxylic acid (TCA) cycle. This upregulates tumour mitochondrial oxidative phosphorylation, improving ATP production.^24^ In addition, the ketones and lactate produced as the end products of fibroblastic glycolysis have been shown to promote the growth and metastasis of breast cancer in an immunodeficient mouse model,^25^ by increasing the transcriptional profile of genes associated with ‘stemness’ in cancer cell lines. This suggests a link between metabolism and cancer stem cells (CSCs, see below), which could potentially be severed using oxidative mitochondrial metabolism inhibitors, such as metformin.^24^ Patient-derived lymphoma CAFs have also been shown to secrete significant amounts of pyruvate, fuelling lymphoma tumours via the reverse Warburg effect.^26^ Patient-derived prostate cancer CAFs can assist nutrient-deprived prostate and pancreatic cancer cell lines, via secretion of exosomes containing intact metabolites, including amino acids and TCA-cycle intermediates, allowing the tumour cells to continue growing rapidly.^27^ Interestingly, tumours can enhance their own survival by secreting exosomes that alter CAF metabolism. In one study, breast-cancer-secreted exosomes containing miR-105 reprogrammed CAFs by activating MYC signalling. This imbued the CAFs with the capacity to enhance glucose metabolism when nutrients were sufficient, or detoxify metabolic wastes into energy-rich metabolites when nutrients were scarce, thus ensuring the tumour remained sufficiently energised for sustained growth.^28^

### ECM remodelling and tumour invasion

The ECM is a dynamic scaffold of glycosaminoglycans and proteins that functions as a cell-docking station and facilitates cell communication, differentiation, adhesion and movement. It is constantly being degraded and synthesised by fibroblasts in a regulated response to changing tissue conditions; degradation is facilitated by matrix metalloproteinases (MMPs) and synthesis is associated with the increased production of proteins, such as collagen.^29^ The ECM also functions as a biological repository, sequestering a wide range of growth factors and cytokines.^30^ CAFs perpetually remodel the ECM, and the degradation of ECM molecules by CAF-generated MMPs not only releases sequestered proteins to create a self-sustaining feed-forward loop of CAF activation and ECM remodelling but also creates new molecule fragments with potential pro-migratory and pro-angiogenic roles.^31,32^ Moreover, ECM degradation physically frees up space for proliferating and migrating tumour cells. The net outcome is accelerated cancer progression. CAFs additionally stimulate constitutive collagen cross-linking; they are the primary source of the cross-linking inducer lysyl oxidase (LOX) in mouse mammary cancer,^33^ and LOX inhibition in a mouse breast cancer model led to a decline in fibrosis, tumour invasion and metastasis.^34^ CAF-derived FAP organises these cross-linked fibres into parallel orientations through its serine protease activity, forming stiff ‘migration highways’ that enhance the directionality and velocity of pancreatic cancer cells during invasion via activation of the β_1_-integrin/FAK signalling pathway; without FAP, the ECM remains disorganised and invasion is impeded.^35^ Lung cancer cells can also adhere to CAFs via integrin α5β1 and quickly migrate along the long fibroblast protrusions, acquiring invasive potential in three-dimensional collagen matrix.^36^ In addition, CAFs can directly adhere to the skin and squamous cell carcinoma (SCC) cells, inducing ECM tracks ahead to further accelerate invasion.^37,38^

### CAFs, cancer stemness and metastasis

CAFs are suggested to play a role in the induction and maintenance of a small population of tumorigenic cells that are capable of self-renewal and multilineage differentiation. These cells, termed CSCs, have a crucial role in cancer initiation, with one CSC capable of giving rise to an entire tumour.^39^ CSCs are predominantly dormant so they are naturally resistant to anti-proliferative chemotherapy and therefore difficult to eradicate, and might be responsible for relapse years after initial treatment.^40^ CAF-derived chemokine (CC motif) ligand 2 (CCL2), IL-6, hepatocyte growth factor (HGF), osteopontin (OPN) and stromal cell-derived factor 1 (SDF-1) each independently induce the CSC phenotype in epithelial cells.^41–43^

CAFs can enhance cancer stemness by inducing an epithelial-to-mesenchymal transition (EMT) programme in cancer cells, as described in a mouse model of human prostate cancer.^44^ EMT is associated with increased motility of cancer cells, with loss of cell–cell adhesion and polarity,^45^ and increased resistance to therapy. IL-6 is one example of a CAF-derived factor that induces EMT in oesophageal cancer, and IL-6 inhibition restored sensitivity of patient-derived oesophageal cancer organoids to chemotherapy and radiotherapy.^46^ During EMT, there is also evidence of a reversion to the epithelial phenotype (mesenchymal-to-epithelial transition, MET), allowing metastatic outgrowth (discussed in ref. ^47^) CAFs are able to accompany disseminated tumour cells in the circulation, providing survival cues and an early growth advantage at the metastatic site; partial depletion of CAFs at this stage significantly decreases the number of successful lung metastases in a mouse model.^48^ In serous ovarian cancer, this phenomenon is a feature of high-grade tumours, where ascetic tumour cells marked by integrin α-5 (ITGA5) expression are prone to forming spheroids with CAFs. When CAFs were pre-treated with imatinib to inhibit PDGFR signalling, mice with ovarian cancer had reduced peritoneal colonisation and improved survival.^49^ Infiltrating tumour cells can also secrete extracellular vesicles containing TGF-β, educating the foreign niche to resemble the primary microenvironment. The activated tissue-resident fibroblasts then express factors to promote epithelial colonisation (MET) and proliferation, initiating secondary tumorigenesis.^50–52^

**Fig. 1:**
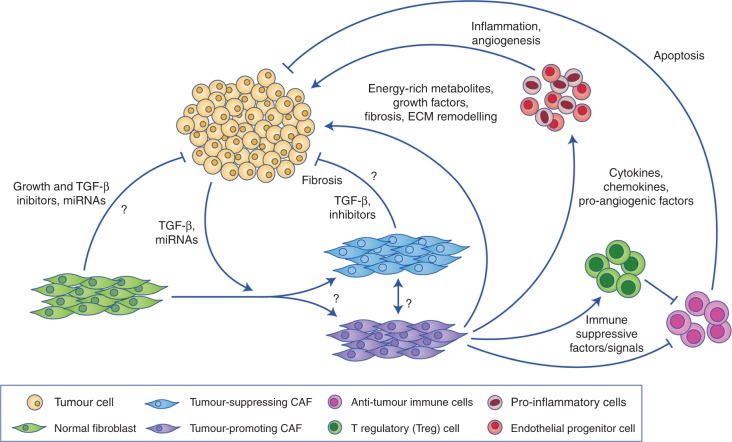
Roles of CAFs in tumorigenesis. Tumour cells and the surrounding fibroblasts or CAFs communicate to generate a microenvironment that either promotes or suppresses tumorigenesis. Normal fibroblasts can be stimulated to become CAFs by the tumour, however, the biological programmes that determine their skew towards a tumour-promoting or tumour-suppressing subtype remain unknown. The tumour-promoting subtypes aid tumour growth, survival and spread both directly and indirectly, through other tumour-associated stromal cell types. Both normal fibroblasts and CAFs have been shown to inhibit or retard tumorigenesis, but the mechanisms are relatively unknown

Tumour-promoting CAFs are therefore at least partly responsible for the acquisition of proliferative, pro-inflammatory, immune-suppressive, angiogenic, pro-invasive and pro-metastatic environments that are required for aggressive tumorigenesis (Fig. 1).

## The tumour-suppressing CAF phenotype

Despite the overwhelming evidence indicating that CAFs have a tumour-promoting role, there are studies suggesting that these cells, along with normal fibroblasts, are involved in tumour suppression. Whether these fibroblast subtypes are normal fibroblasts that are resistant to conversion into CAFs, or distinct anti-tumour CAF subpopulations, remain unknown (Fig. 1).

The notion that normal cell types have tumour-suppressing properties was demonstrated by injecting a teratoma cell line into an early mouse embryo; the resulting postnatal animals were derived from both host and transplanted tumour cells, but the tumorigenic phenotype had somehow been suppressed so that normal development ensued.^53^ Normal, resting fibroblasts were suggested to be the tumour-suppressing cell type, as they were shown to restrain the growth of transformed baby hamster kidney cells in vitro^54^ and transformed keratinocytes in vivo*.*^55^ Another in vivo study showed that PDGFRα^+^ CAFs isolated from a PDAC mouse model stimulated subcutaneous PDAC tumour growth in mice by 76%, while normal pancreatic fibroblasts inhibited tumour growth by 65%.^56^ Tumour growth-suppressive factors, such as whey acidic protein four-disulphide core domain 1 (WFDC1), were highly expressed in resting fibroblasts but downregulated in CAFs,^57^ suggesting that dormant fibroblasts might indeed suppress tumorigenesis. Resting fibroblasts at metastatic sites can also impede colonisation, as normal bone marrow stroma was shown to induce quiescence in infiltrating breast cancer cells via the gap junction-mediated transfer of several miRNAs targeting CXCL12.^58^

Studies have also uncovered the presence of tumour-suppressing fibroblasts in established tumours. PDAC is notorious for its large fibrotic tumour response, with the majority of tumour volume being composed of α-SMA^+^ fibroblasts.^8^ Depleting these fibroblasts was hypothesised to reduce PDAC aggression, but clinical studies relying on this strategy revealed accelerated disease progression.^59^ Upon further investigation in mice, it was confirmed that deletion of α-SMA^+^ fibroblasts induced invasive tumours that showed enhanced EMT and increased numbers of CSCs, and resulted in reduced survival. This outcome was primarily due to a decline in immune surveillance and increased infiltration of Treg cells. The fibrotic response associated with PDAC is therefore a host defence mechanism against tumour invasion.^8^ Unexpected results were also seen with alteration of Hh signalling in CAFs. It has been shown that tumour-derived sonic hedgehog (Shh) activates smoothened (SMO) in fibroblasts, which activates the fibroblasts and consequently promotes PDAC desmoplasia.^60^ When Hh signalling was inhibited in CAFs in a PDAC mouse model using the drug IPI-926, the tumours became transiently vascularised and delivery of the chemotherapeutic agent gemcitabine was enhanced, increasing mouse survival. This study demonstrates another example of how CAFs can impact tumour chemoresistance, as the desmoplastic response causes poor intratumoural vascularity and consequently inefficient drug delivery.^61^ Thus, naturally, it was hypothesised that continued research into Hh-signalling inhibition would be therapeutically advantageous. This was not always the case, as deletion of Shh enhanced disease progression and reduced CAF content in a PDAC model,^9^ and activating Hh signalling in fibroblasts markedly reduced tumour load and disease progression in a colon cancer model.^62^ Hh-signalling CAFs therefore appear to act as both tumour promoters and tumour suppressors, with the specific factors that predict this behaviour across multiple cancer types remaining unknown. Interest surrounding serum amyloid A (Saa3) expression in mouse Pdgfrα^+^ CAFs has also recently come into light, as Pdgfrα^+^Saa3^+^ CAFs were shown to stimulate PDAC growth in an orthotopic mouse model while Pdgfrα^+^Saa3^−^ CAFs inhibited growth.^56^ Inhibiting Saa3 could therefore result in an abundance of anti-tumour CAFs, but the initial findings of this study await further validation as to their suitability as a therapeutic target for PDAC. Interestingly, the phenomenon of CAF-induced cancer regression was demonstrated in a recent study, whereby CAFs were shown to restore lung SCC spheroids from a dysplastic to hyperplastic state through the suppression of high epithelial Sox2 activity. As such, CAFs may have the ability to override cell intrinsic oncogenic changes and determine the extent of disease progression.^63^

There is a growing body of evidence suggesting that cancer cells influence whether or not fibroblasts suppress tumour growth. In the normal setting, stromal cells express TGF-β inhibitors (reviewed in ref. ^64^) and low expression of one such fibroblast-derived inhibitor, asporin, is correlated with poor survival in breast cancer patients. However, asporin expression appears to be influenced by the tumour’s genetic subtype, suggesting that the level of fibroblast-induced tumour suppression can be partly determined by the tumour itself.^65^ Likewise, breast tumours can downregulate the roundabout homologue 1 (Robo1) receptor to stop the tumour-suppressive effects of its fibroblast-derived ligand, slit homologue 2 (SLIT2), which inhibits proliferative β-catenin signalling. This change in expression is associated with poor prognosis.^66,67^

The tumour-suppressive phenotype of fibroblasts and CAFs is therefore dependent on the factors they produce, which remain largely uncharacterised. Successful tumours must then be able to evolve mechanisms to control, evade or overcome fibroblast-induced suppression.

## Therapeutic implications

Because cancer is a complex, dynamic and adaptive ecosystem reinforced by genetic diversity and epigenetic plasticity, the rapid emergence of drug resistance is problematic for many patients. Consequently, there is a need to investigate therapies that are not necessarily directed at the tumour cells themselves and, as such, CAFs provide an attractive target: they are genetically stable and consequently less likely than tumour cells to acquire therapeutic resistance, and their presence across different cancer types entices a broad applicability of effective therapies. Some studies have examined the effect of ablating the prominent CAF subtypes on carcinogenesis: deletion of FAP-expressing fibroblasts induced rapid tumour necrosis in LLC^14^ and impeded metastasis in PDAC,^68^ whereas elimination of α-SMA fibroblasts enhanced tumorigenesis in both mouse and clinical models.^8,59^ CAF-directed therapy should therefore be designed against specific pro-tumour factors or functions with the aim of preventing CAF function or activation, or reprogramming CAFs back into a normal, resting phenotype.

### Targeting CAFs as a method of cancer therapy

FAP has been the focus of many CAF-directed preclinical and clinical cancer studies. A DNA vaccine directed against FAP was shown to significantly suppress primary and secondary tumours in a murine colon cancer model by upregulating cytotoxic T-cell-mediated killing, increasing animal survival.^69^ Tumour angiogenesis was also suppressed following depletion of FAP^+^ CAFs via FAP-targeted chimeric antigen receptor (CAR) T cells, with resultant murine pancreatic tumour retardation.^70^ The enzymatic activity of FAP has also been inhibited using the drug PT-100, which attenuated tumour growth in many mouse models,^71^ but showed minimal activity in phase 2 trials of patients with metastatic CRC.^72^ The humanised anti-FAP antibody, sibrotuzumab, also showed no beneficial effect in phase 2 trials for metastatic CRC.^73^ In both of these trials, the patients were heavily pre-treated and could therefore represent a refractory patient population. It is also hypothesised that the biological effects of FAP may be more prominent in smaller tumours or earlier stages of CRC, not in late-stage metastasis. Other CAF subtypes, namely *CD10*^+^*GPR77*^+^ and *IL-7*^+^ CAFs, have been targeted pre-clinically, resulting in impaired tumour stemness and growth with restored chemosensitivity in breast cancer patient-derived xenografts (PDXs) and cell lines, respectively.^74,75^ Intriguingly, *CD10*^+^*GPR77*^+^ CAFs directly conveyed a chemo-resistant phenotype on breast cancer cells in co-culture, co-transplant and PDX models through the secretion of factors such as IL-6 and the upregulation of the multidrug-resistance transporter ABCG2.^74^ The SOM230 analogue, Pasireotide, has been used in mouse models of PDAC to prevent IL-6 synthesis (amongst other proteins) through activation of the somatostatin (sst1) receptor, which then inhibits the mammalian target of the rapamycin (mTOR)/4E-BP1 pathway. This pathway is highly activated in α-SMA^+^ CAFs isolated from human PDAC resections, and when SOM-230 was administered to mice engrafted with human PDAC there was a reduction in tumour growth and chemoresistance to gemcitabine. This drug targets the pathway in CAFs specifically, as the sst1 receptor is not present in the epithelium.^76^ CAF-mediated secretion of IL-6 has also been inhibited by resveratrol, inducing a switch in expression from N-cadherin to E-cadherin in migrating mouse cholangiocarcinoma cells to diminish their invasion and induce autophagy.^77^

Additional targets for cancer treatment are MMPs, even though they are not solely expressed by CAFs, and many different MMP inhibitors have been developed and tested in phase 1, 2 and 3 clinical trials. Unfortunately, all clinical trials testing small molecule, broad-spectrum MMP inhibitors in a range of cancer types and stages, to date, have failed to improve clinical outcomes and resulted in major musculoskeletal toxicity in many cases. This field is advancing tremendously, however, with the development of many small molecule inhibitors and antibodies targeting specific domains of particular tumour-promoting MMPs, and improved targeting of natural MMP inhibitors, such as the tissue inhibitors of metalloproteinases (TIMPs). This topic is extensively reviewed in ref. ^78^ Recent clinical trials combining gemcitabine with the SMO inhibitor, vismodegib, have also been underway in patients with metastatic PDAC, despite the preclinical evidence suggesting Hh-driven PDAC CAFs are also tumour suppressors;^9^ fibrosis was reduced in 45.4% of patients in a pilot trial, and no toxicity was observed in a phase 1b/2 trial, but there was no improved overall response rate or survival with dual therapy in either trial.^79,80^ Perhaps these disappointments are due to the non-selective ablation of Hh-driven, tumour-suppressing CAF subtypes amongst the tumour-promoting subtypes. However, 3 of 12 patients with metastatic triple-negative breast cancer treated with docetaxel and the SMO inhibitor Sonidegib in a recent phase 1 clinical trial showed clinical benefit, with one experiencing a complete response. The accompanying preclinical data suggest that mechanistically these outcomes were due to the downregulation of CSC markers.^81^ As more is learnt about the heterogeneity of Hh-signalling CAFs, the aim is to target only the pro-tumour subsets and ultimately lead to improved cancer therapies.

Methods to inhibit CAF activation are also in development as novel cancer therapeutics. The downregulation of the androgen receptor (AR) in dermal fibroblasts was shown to induce early CAF activation and enhance tumorigenicity of SCC and melanoma cells. As such, this study suggested the use of bromodomain (BET) inhibitors, such as JQ1, to restore AR expression. This resulted in reduced CAF activity and prevented tumour growth in a melanoma mouse model.^82^ Compounds such as the synthetic vitamin-D derivative, calcipotriol, have also been used to block the TGF-β-mediated differentiation of pancreatic fibroblasts into CAFs, decreasing inflammation and fibrosis and improving chemotherapeutic responsiveness in mice with spontaneous PDAC.^83^ Similarly, the endogenous bioactive lipid lipoxin A4 (LXA4) was used to inhibit Smad2/3 signalling in pancreatic fibroblasts to ultimately retard murine pancreatic tumour growth.^84^ In other approaches, the epigenetic machinery inducing CAF activation has been targeted. Firstly, TGF-β-mediated CAF differentiation was targeted by using Scriptaid to inhibit histone deacetylase. Reduced fibroblast content and activity, impaired collective CAF-tumour cell invasion and delayed melanoma growth in vivo resulted.^85^ Secondly, targeting the RNA editor, Adenosine Deaminase family acting on RNA (ADAR1), appears warranted in CRC; ADAR1 is overexpressed in human CRC and leads to elevated antizyme inhibitor 1 (*AZIN1*) RNA editing levels, which is correlated with increased CAF marker expression and aggressive tumours.^86^

Reprogramming activated fibroblasts or CAFs back into their dormant state is another strategy for impairing tumorigenesis. miRNAs have attracted interest in this context, and in the case of lung fibrosis, upregulation of the miR-19a-19b-20a sub-cluster was observed during disease progression. This sub-cluster suppressed TGF-β-induced fibroblast activation, suggesting that normal fibroblasts attempt to stop their own activation.^87^ Upregulation of the TGF-β pathway inhibitor, miR-145, was also observed in TGF-β-induced and primary oral cancer CAFs. When normal oral fibroblasts were transfected with miR-145 prior to TGF-β treatment, their in vitro activation was impaired, and when transfected following treatment, their quiescent phenotype was rescued. Thus, it is again seen that normal fibroblasts may inherently contain a negative feedback loop to protect against CAF conversion.^88^ Another study demonstrated that CAFs within human breast tumours had decreased levels of the tumour suppressor miRNA, Let-7b, compared with their normal fibroblast counterparts. Inhibition of Let-7b in these normal breast fibroblasts increased their activation and capacity to induce breast cancer cell EMT in vitro, and enhanced tumour growth in a murine breast cancer model. The opposite was observed upon re-expression of Let-7b in human breast CAFs, and the cancer-promoting abilities of breast myofibroblasts were reduced.^89^ Pancreatic CAFs have also been reprogrammed towards quiescence via activation of the tumour-suppressor p53 by the Nutlin-3a derivative RG7112, resulting in a reduction of murine pancreatic desmoplasia.^90^ The combined inhibition of DNA methyltransferase activity and Janus kinase (JAK) signalling also resulted in the long-term phenotypical and molecular reversion of activated CAFs to normal, resting fibroblasts, causing murine breast tumours to become less invasive.^91^

Although some preclinical studies have demonstrated the benefits of targeting CAFs alone, presumably the simultaneous targeting of both the tumour and CAF compartments will ultimately improve efficacy in clinical trials. Some preclinical studies have targeted both the tumour and CAFs with one therapeutic agent. One group designed a novel dendrimer conjugated to the mTOR inhibitor rapamycin to successfully inhibit mTOR signalling and VEGF expression in prostate cancer cells and fibroblasts, and reduce fibroblast-mediated prostate tumour progression and metastasis.^92^ Another group targeted neuregulin (NRG1), which is highly expressed in the cancer cells and CAFs of PDAC. Their NRG1 inhibitory antibody, 7E3, was able to promote apoptosis in the pancreatic tumour cells and CAFs, and inhibit the growth of the tumour.^93^ Other studies have combined different CAF-targeting and tumour-targeting drugs to investigate their dual anti-tumour efficacy. For example, compromising the integrity of collagen fibres using nab-paclitaxel, combined with gemcitabine, reduced the number of CAFs and improved overall survival in a phase 3 clinical trial of pancreatic cancer, compared with either agent alone.^94,95^ Similarly, in the preclinical setting, only the dual administration of the chemotherapeutic oxaliplatin with PT-100 increased mouse survival by decreasing CAF content, colon tumour growth, angiogenesis and pro-tumour immune cell recruitment.^96^ This is not always the outcome, however, as PT-100 combined with cisplatin for stage IV melanoma or with docetaxel for non-small-cell lung carcinoma (NSCLC) did not show a therapeutic advantage in phase 2 human trials.^97,98^ Other trials have attempted to reduce tumorigenesis by disrupting the cross-talk between the cancer and CAFs. As an example, HGF is predominantly produced by CAFs and activates its cognate receptor, c-Met kinase, on tumour cells, promoting tumorigenesis and chemoresistance via a variety of mechanisms (reviewed in ref. ^99^). HGF and c-Met have both been targeted in many preclinical and clinical trials; for example, inhibition of HGF via a neutralising antibody or siRNA knockdown impaired gastric tumour growth in mice,^100^ and the combination of gemcitabine with the c-Met inhibitor, tivantinib, demonstrated early signs of anti-tumour activity in various solid tumours in a phase 1 trial, warranting a phase 2 trial.^101^ In recent times, many c-Met small molecule inhibitors have been in development and they are reviewed in ref. ^102^

There are many tumour-CAF co-targeting clinical trials currently recruiting patients: for example, RO6874281 is a recombinant fusion protein that targets an engineered, variant form of IL-2 to human FAP^+^ cells, to stimulate a local immune response and therefore improve anti-tumour immunity. It is currently being combined with trastuzumab (for HER2^+^ breast cancers) or with the epidermal growth factor receptor (EGFR) inhibitor cetuximab (for head and neck cancers) in phase 1 trials (NCT02627274), or with the anti-PD-L1 antibody atezolizumab in a phase 2 trial investigating advanced and/or metastatic solid tumours (NCT03386721). The scientific community awaits the outcomes of these, and other, tumour-CAF-targeted trials.

### Using CAFs as a means of drug delivery

Another strategy for CAF-directed anti-cancer therapy involves the skewing of CAFs towards a suppressive phenotype. However, to the best of our knowledge, no studies have directly explored this approach; the lack of specific biomarkers for CAFs and for their tumour-promoting and tumour-suppressing phenotypes has become a hindrance. However, one way to evade the issues surrounding CAF heterogeneity (for now) involves the use of the general CAF population as a drug delivery tool. A prodrug has been designed to be cleaved, using the protease activity of FAP, into the cytotoxic drug thapsigargin, and it produced a local therapeutic response in mouse models of breast and prostate cancer.^103^ MMPs have also been used to cleave MMP-sensitive micelles encapsulating the chemotherapeutic drug docetaxel, releasing it locally to reduce cervical cancer growth in mice.^104^ Moreover, nanoparticles containing plasmids encoding a secreted form of the pro-apoptotic factor TRAIL (sTRAIL) have been non-specifically targeted to CAFs, modifying them to express sTRAIL. This approach killed nearly all of the local bladder and pancreatic tumour cells and diminished CAF numbers, prolonging survival and efficiently ameliorating metastasis in preclinical models.^105^ Leucine-rich-repeat-containing 15 (LRRC15)^+^ CAFs have also been targeted using a LRRC15-antibody drug conjugate (ABBV-085) to deliver the potent anti-mitotic drug monomethyl auristatin E (MMAE) in PDX models of NSCLC-adenocarcinoma, osteocarcinoma, breast cancer and glioblastoma, significantly reducing tumour volume. Nevertheless, complete responses in these models were only seen when combined with various types of chemotherapy, radiation and immune therapy agents.^106^

Another approach still in its infancy uses CAFs and CAF-derived factors as ecological traps, designed to mimic the normal niche to attract disseminated cancer cells that are subsequently killed via methods, such as TRAIL-induced apoptosis and trap-targeted stereotactic radiation.^107^ However, this method involves surgical implantation, creating a wound site that in itself was observed to activate tissue-resident fibroblasts and attract more cancer cells.^107^ One group was able to encapsulate live CAFs in microparticles that could then be intraperitoneally injected, without wounding. The CAF-derived ECM surface of the beads selectively captured disseminated cells from different tumour origins, and the iron oxide nanoparticles incorporated into the bead surface allowed for magnetic retrieval of the microparticles. This technique was able to prolong mouse survival by delaying peritoneal metastasis.^107^

### CAFs as prognostic tools

The differential expression of specific genes in the tumour stroma, compared with the normal stroma of the same tissue, generates expression signatures that can offer prognostic clues in many different types of cancers. One of the first studies to recognise the prognostic relevance of the tumour stroma to survival and recurrence, in breast cancer patients, identified a 26-gene ‘stroma-derived prognostic predictor’.^108^ This signature, however, was not specific to CAFs but to all cells in the tumour stroma. Other studies have since derived prognostic gene signatures from the CAF population specifically, for example in CRC,^5^ NSCLC^109^ and hepatocellular carcinoma.^110^ However, the CAF-associated signatures identified in these studies do not overlap; for example, the CRC 4-gene signature includes *FAP* and *CALD1* (the gene encoding caldesmon 1),^5^ the 11-gene NSCLC signature includes *THBS2* (the gene encoding thrombospondin 2) and *CLU* (the gene encoding clusterin),^109^ and the 12-marker liver cancer panel includes fibroblast growth factor 5 (FGF5) and MMP1.^110^ Again, heterogeneity is to blame for the lack of a uniform CAF-associated prognostic gene signature, and it may be that every different type of cancer has its own specific predictor. One group has also found a CAF-associated gene signature that predicts chemotherapy resistance in the neoadjuvant setting for breast cancer, based on the differential expression of 50 genes, including *DCN* (the gene encoding decorin), *COL1A2*(the gene encoding collagen type 1α2), *FAP*, *CALD1* and *THBS2.*^111^In addition, the presence of CD146-expressing CAFs predicts tamoxifen sensitivity and better treatment outcome in patients with oestrogen receptor-positive (ER^+^) breast cancer, as they maintain ER expression (unlike CD146-null CAFs).^112^

Tumour and CAF data can be obtained from the blood and peritoneal fluid, and sequential liquid biopsy samples allow the dynamic monitoring of these cells during cancer progression. This technique was originally used to detect disseminated cancer cells, which were indicative of increased recurrence and poorer survival and therefore served as prognostic, metastatic markers.^107^ However, this technology was subsequently enriched to detect circulating CAFs, demonstrating that CAFs were present in 88% of breast cancer patients with metastases, 23% of patients with localised disease and 0% of healthy donors.^113^ Moreover, in oesophageal cancer, ADAM12 is the serum-borne marker for IL-6^+^ CAFs, and its presence predicts poor response to neoadjuvant chemoradiation.^46^

## CAF markers and heterogeneity

Historically, research has underestimated the complexity of CAF heterogeneity and studies have used the entire, mixed, CAF population to draw general conclusions, an approach that is likely to have resulted in observational variability and ultimately enhanced confusion in the field. As we come to appreciate the complexity of CAFs, studies are now attempting to single out specific CAF subtypes, predominantly targeting the two most common types— either α-SMA^+^ or FAP^+^ CAFs. But, even this approach has had variable results, and their co-expression is also debatable. One study demonstrated that they define completely different CAF subsets, at least in CRC, with α-SMA associating with other activated fibroblast markers such as transgelin (TAGLN) and platelet-derived growth factor subunit A (PDGFA), whilst FAP associated with other markers, including DCN and COL1A2.^10^ It is worth noting that this was the first comprehensive study that attempted to define human CAF subsets, using single-cell sequencing. Another study defined α-SMA^High^FAP^+^ pancreatic CAFs as a myofibroblastic, active subtype responsive to TGF-β, while the remaining α-SMA^Low^ CAFs were shown to secrete inflammatory mediators such as IL-6 that promoted the growth and proliferation of patient-derived PDAC organoids. These two CAF subtypes were mutually exclusive, but reversible in different culture conditions.^114^ Furthermore, another study that defined four breast CAF subsets, based on their expression of α-SMA, FAP, FSP1, PDGFRβ and CD29, demonstrated that α-SMA^High^FAP^High^ CAFs were associated with an immune-suppressive environment, enhancing Treg cells via CXCL12 secretion. The α-SMA^High^FAP^Neg^ CAF subset was devoid of these properties.^115^ These last two studies are some of the first to examine the potential functional roles of different CAF populations in pancreatic and breast cancer, respectively, yet the α-SMA^+^FAP^+^ CAF subset they both identified had slightly different properties. Similar CAF subtypes may therefore have unique roles in each tissue type, adding an extra layer of complexity. There are also differences in CAF marker expression between tissues; for example, 43.5% of α-SMA^+^ fibroblasts co-expressed FSP1 in pancreatic cancer, but this overlap was reduced to 10.9% in breast cancer.^11^ CAFs may therefore be further regulated by other unknown, tissue-specific factors.

Other studies have attempted to define CAF heterogeneity, not based on α-SMA and FAP expression. Bartoschek et al.^116^ were able to define three distinct populations of breast cancer CAFs from a mouse model, which was confirmed in patients; vascular CAFs (vCAFs) were Nidogen2^+^, matrix-related CAFs (mCAFs) were Pdgfrα^+^ and developmental CAFs (dCAFs) were Pdgfrβ^−^Scrg1^+^. αSMA and FAP expression did not segregate with these populations. The presence of vCAFs and mCAFs correlated with metastatic dissemination, and the mCAF signature additionally correlated to a treatment-predictive stromal signature in breast cancer. Each subset could be spatially separated and this was linked to different origins; vCAFs from perivascular cells, mCAFs from resident fibroblasts and dCAFs from tumour cells that had undergone EMT.^116^ The heterogeneity of CAFs is understandable given different source populations in different locations. Another example of spatially demarcated CAFs was demonstrated by Cremasco et al.^117^ in a mouse model of immune-excluded breast cancer, where FAP^+^PDPN^+^ CAFs were localised around the tumour edge and in close contact with T cells, while FAP^+^PDPN^−^ ‘cancer-associated pericytes’ (CAPs) surrounded vessels. Interestingly, CAFs were enriched in TGF-β signalling and fibrosis and produced nitric oxide to suppress T-cell proliferation; CAPs were not immunosuppressive.^117^ Moreover, Biffi et al.^118^ demonstrated that myofibroblastic CAFs (myCAFs) were found adjacent to PDAC tumours, while inflammatory CAFs (iCAFs) were preferentially localised in the dense PDAC stroma. This study demonstrated another concept alluded to above, that tumours can influence CAF activation and as a result also CAF heterogeneity; PDAC tumour-derived IL-1 steered CAFs towards an iCAF phenotype, while TGF-β biased towards myCAFs. CAFs in different locations could therefore have been exposed to different tumour ligands, influencing their phenotype. TGF-β was also shown to convert iCAFs into myCAFs, via antagonism of the JAK/STAT pathway and downregulation of IL-1R1 expression.^118^ The presence of interconvertible myCAFs and iCAFs in PDAC is supported by Ohlund et al.^114^ Biffi et al.^118^ therefore suggest drug combinations targeting both iCAFs and myCAFs, to decrease tumour-promoting inflammation and deplete the dense stroma impeding drug delivery. In another study, Neuzillet et al.^119^ proposed the ‘pCAFassigner' classification system to segregate their three defined human-derived PDAC CAF subtypes based on the expression of periostin (POSTN), myosin-11 (MYH11) and PDPN. Tumour-conditioned medium was shown to induce two of the three CAF subtypes in pancreatic stellate cells in vitro, suggesting tumours can influence dynamic CAF states.^119^ It also appears that the cancer stage can influence the dominating CAF phenotype, via cancer-derived exosomes, particularly in CRC; early-stage CRC exosomes promoted highly proliferative and angiogenic CAFs, while late-stage exosomes from metastatic CRC lines induced highly invasive CAFs.^120^

## Conclusion

Tumours and their local microenvironment form a complex ecosystem that can ultimately promote cancer progression. It has now become widely accepted that CAFs can have a dual role in tumorigenesis, owing to their heterogeneity; they can either promote cancer via induction of an inflammatory, immune-suppressive, angiogenic, energy-rich, invasive and metastatic environment, or suppress cancer via predominantly unknown mechanisms (Fig. 1). It might be that fibroblasts begin as a tumour-suppressive cell type, but as the tumour develops it begins to influence the environment and transforms these fibroblasts into pro-tumoural factories. It is therefore conceivable that fibroblasts act as both heroes and villains.

**Fig. 2:**
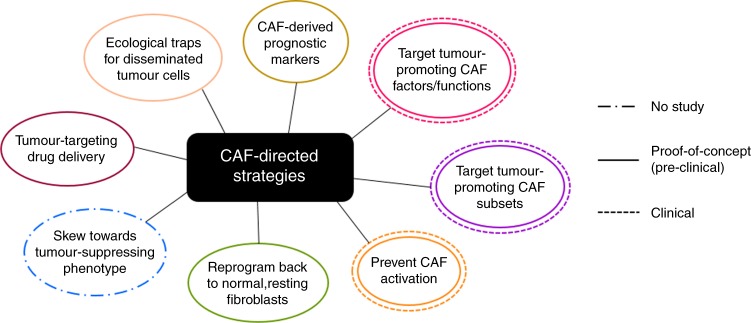
CAF-directed cancer therapies. Outlined are several preclinical and clinical approaches aimed at targeting CAFs in an attempt to impede tumorigenesis. Ideally, for full efficacy, these strategies will be used in conjunction with drugs that target tumours

As different CAF expression profiles may define their role in tumorigenesis, CAF molecular marker combinations must be further refined to assist in the classification of pro- and anti-tumour subsets in different tissues. This will provide us with better prognostic CAF biomarkers and more specific avenues for CAF-directed cancer treatments, allowing us to precisely target the tumour-promoting subtypes and CAF-derived factors or skew the CAF phenotype away from tumour promotion, or prevent CAF activation altogether (Fig. 2). These novel strategies can then be combined with other tumour-targeting drugs to ultimately improve treatment efficacy and patient survival.

## Data Availability

Not applicable.
